# Effects of Artificial Flooding for Hydroelectric Development on the Population of *Mansonia humeralis* (Diptera: Culicidae) in the Paraná River, São Paulo, Brazil

**DOI:** 10.1155/2012/598789

**Published:** 2012-02-29

**Authors:** Marcia Bicudo de Paula, Almério de Castro Gomes, Delsio Natal, Ana Maria Ribeiro de Castro Duarte, Luís Filipe Mucci

**Affiliations:** ^1^Departamento de Epidemiologia, Faculdade de Saúde Pública, Universidade de São Paulo, Avenida Dr. Arnaldo 715, 01246-904 São Paulo, SP, Brazil; ^2^Laboratório de Bioquímica e Biologia Molecular, Superintendência de Controle de Endemias, Rua Paula Souza, 166 - 5° andar, 01027-000 São Paulo, SP, Brazil; ^3^Laboratório de Culicídeos, Superintendência de Controle de Endemias, Praça Coronel Vitoriano 23, 12020-020 Taubaté, SP, Brazil

## Abstract

The closure of two phases of the dam at the Porto Primavera Hydroelectric Plant on the Paraná River flooded a flawed system located in the Municipality of Presidente Epitácio, São Paulo state, favoring the proliferation of aquatic weeds. This study aimed to observe the population of *Mansonia humeralis* in the area, monitoring the richness, diversity, and dominance of this species both before and during different phases of reservoir flooding as well as evaluate its possible consequences concerning human and animal contact. Adult mosquitoes were collected monthly in the following periods: at the original level, after the first flood, and after the maximum level had been reached between 1997 and 2002. Collection methods used were an aspirator, a Shannon trap, and the Human Attractive Technique. A total of 30,723 mosquitoes were collected, *Ma. humeralis* accounting for 3.1% in the preflood phase, 59.6% in the intermediate, and 53.8% at maximum level. This species is relevant to public health, since the prospect of continued contact between *Ma. humeralis* and the human population enhances the dam's importance in the production of nuisance mosquitoes, possibly facilitating the transmission of arboviruses. Local authorities should continue to monitor culicid activity through sustainable entomological surveillance.

## 1. Introduction

Anthropic changes to the natural environment invariably impact biodiversity, and new habitats often become available after landscape alterations, particularly among culicids [1]. According to Forattini [2], this dynamic favors selective processes. Newly established ecotopes promote the proliferation of mosquito species that become nuisances or disease vectors for humans [3].

Female mosquitoes of the family Culicidae oviposit in several types and dimensions of reservoir water, where they cooccur with other species and are associated with plant matter. During immature stages, mosquitoes of the genera *Mansonia* Blanchard and *Coquillettidia* Dyar affix themselves to the roots of aquatic macrophytes, from which they derive oxygen accumulated in the aerenchyma of the plant floating organs [4].

The eleven *Mansonia *species listed for Brazil [5, 6] depend on macrophytes during larval and pupal stages. As with most culicids, only adult females require blood meals (for oocyte maturation). Mosquitoes from the genus *Mansonia* are a nuisance to humans and animals in situations of high density and can become a public health concern due to their anthropophilic behavior [7].

Our group hypothesized that the damming of the Paraná River and construction of the Engenheiro Sérgio Motta Hydroelectric Plant, more commonly known as the Porto Primavera Hydroelectric Plant (PPHP), produced a favorable environment for mosquitoes of the tribe Mansoniini owing to the propagation of macrophytes. The present study aimed to monitor the richness, diversity, and dominance of the *Ma. humeralis* Dyar and Knab population in different phases of reservoir flooding and assess its possible consequences regarding human and animal contact. Data concerning other species are also presented.

## 2. Materials and Methods

The present research was undertaken on the banks of the PPHP reservoir, formed by the damming of the Paraná River, 28 km upstream from its confluence with the Paranapanema River. Although the reservoir impacts several municipalities in Mato Grosso do Sul [8, 9] and São Paulo states [10], the study area was located in the Presidente Epitácio Municipality in São Paulo state, part of a larger project developed in this area.

Prior to the establishment of the PPHP, the study region originally comprised a complex of flooded areas known as the “Lagoa São Paulo Ecological Reserve”, previously formed by the São Paulo, Bonita, Comprida, Tremendal, and Jota lakes and consisting of a mosaic of rearing environments during rainy periods, with accumulated water [11].

After construction of the dam, both permanently submerged and seasonally flooded areas were inundated by the reservoir. The original level of the Paraná River was 247 m above sea level. Flooding was carried out in two stages: the initial phase in January 1999 raised the water level to 253 m, and in March 2001, the second flooding stage elevated waters to the current level of 257 m.

**Figure 1:**
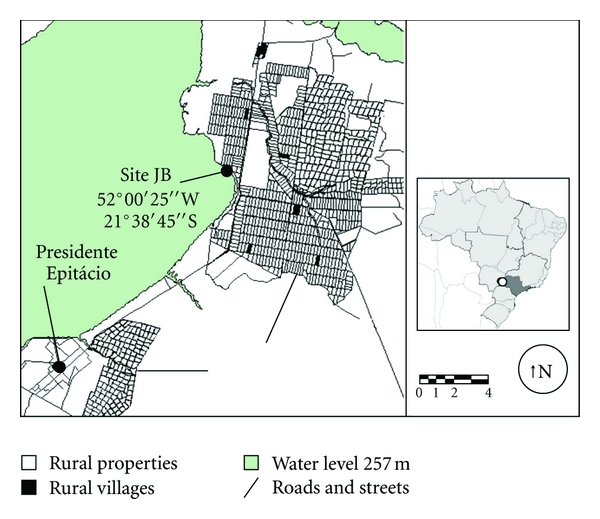
Site JB localization according to PPHP reservoir at water level of 257 meters upper sea level and distinct types of human distribution (rural properties, rural villages, and urban area). Cartographic data: CESP/ITESP/INCRA.

The area designated for mosquito capture, denominated site JB (52°00′25′′W/21°38′45′′S), is composed of flooded lowland, with the presence of floating macrophytes (*Eichhornia crassipes *(Mart.) Solms*, Pistia stratiotes *Linnaeus*, Salvinia *sp. Séguier*, Ricciocarpus natans *(L.) Corda*, Hidrocotyle umbellata *Linnaeus, *Cladium sp.* P. Browne*, Cyperus sp.* Linnaeus*, Egeria najas *Planch), in addition to rural plateau areas containing seven human settlements, with six aggregations of homes (Bairro Campinal and rural villages of Reassentamento Lagoa São Paulo [8]). Residents of these villages and rural landowners come into contact with the reservoir through leisure and/or fishing (Figure 1). Fragments and strips of remnant primitive semideciduous seasonal forest vegetation still occur.

Climate in the region is classified as Aw-Tropical, with a dry winter according to the Köppen system [8]. Average annual precipitation varies from 1,000 to 1,400 mm. Soil type is dark red latossoil/sandy phase with low rainwater infiltration capacity [11].

Culicids were collected during each of the three flood stages: 247 m (07/1997 to 09/1998), 253 m (07/1999 to 09/2000), and 257 m (07/2001 to 09/2002). Adult mosquito collections were undertaken one day per month, using four capture methods: Mechanical aspiration (MA) in one 15 min sampling effort per capture, in the morning period in riparian areas; and Shannon Trap (ST), for 20 min of unit time in the evening at twilight, with intervals as follows: first and second precrepuscular, crepuscular, and first postcrepuscular. There were two individual collectors.

In order to attract a greater number of mosquitoes for anthropophilic behavior, two collection methods were performed using humans: the Human Attractive Technique (HAT 24 h), involving collections over a period of 24 h, with hourly separation of the material gathered by two individual collectors; and the Human Attractive Technique (HATet), consisting of collections in the evening at twilight for 20 min of unit time, with increments divided into first and second precrepuscular; crepuscular; and first, second, third, and fourth postcrepuscular. There were two individual collectors. The last two capture methods were not used during the 257 m stage because of operational and infrastructural difficulties. Field collectors used personal protection to avoid bites.

Mosquitoes were identified in the Entomology Laboratory at the Public Health Faculty of the University of São Paulo.

Samples that were not *Ma. humeralis* were pooled into the category “other species” since our aim was to specifically evaluate the population of this taxon. Frequency of the species investigated was compared to “other species” at stages 247 m, 253 m, and 257 m.

Abbreviations used for species names were in accordance with Reinert [12] and identifications were determined following Forattini [13] and Lane [14].

Variations in temperature and rainfall were correlated with mosquito frequencies for each capture method. Monthly precipitation (mm) and air temperature (°C) data were obtained from the São Paulo Energy Company (CESP) and the Integrated Center of Agro-Meteorological Information, of the Campinas Agronomy Institute (CIIAGRO-IAC).

Statistical analyses were conducted on the mean monthly values of richness [15], diversity (Margalef) [16], and dominance (Berger-Parker) (exclusively for *Ma. humeralis*) [17]. Means between the periods before and after flooding were tested in order to evaluate frequency differences between *Ma. humeralis* and other culicids (*P* ≤ 0.05). Comparative analysis was performed using Mann-Whitney, ANOVA, and Post Hoc (Tukey) tests for indices of richness, diversity, and dominance and comparison of means, with SPSS computer software, version 10.

## 3. Results 

A total of 30,723 culicids were collected during the study, 22,181 were captured at flood stage 247 m (preflooding), and of these, *Ma. humeralis* accounted for 3.1% (695). At the 253 m flooding stage (first flooding), 7,982 mosquitoes were captured, 4,754 (59.6%) of which were *Ma. humeralis*. A total of 560 specimens were collected during the second flooding stage (257 m), *Ma. humeralis* corresponding to 53.8% (301) (Table 1). 

During preflooding, mean monthly richness and diversity values varied among the capture techniques, as did the dominance of *Ma. humeralis* (Table 1). 

Compared to *Ma. humeralis*, the mean richness and diversity values of other culicids decreased after the first flooding stage (247 m–253 m levels) (MA: richness *P* = 0.08, diversity *P* = 0.04, dominance absent; ST: richness *P* = 0.05, diversity *P* = 0.06, dominance *P* = 0.002; HAT 24 h and HATet: richness and diversity: *P* < 0.001, dominance *P* = 0.001). *Ma. humeralis* was not collected using the MA technique during stage 247 m. Following the second flooding stage (253 m–257 m levels), these lower richness and diversity values were maintained (MA: richness and diversity *P* = 0.01, dominance *P* = 0.3; ST: richness *P* = 0.01, diversity *P* = 0.3, dominance *P* = 0.04). Although *Ma. humeralis* dominance remained high in MA captures, samples obtained with ST were substantially reduced (Table 1). 

 Monthly richness and diversity values with the MA capture method were significantly different in comparisons between flood stages, except for richness between flood stage 247 m and 253 m. Means for ST captures were also reduced between flood stages. Richness and diversity values were statistically different in all comparisons, except for diversity between stages 247 m and 253 m, and 253 m and 257 m. Captures using HAT 24 h and HATet yielded significant declines in richness and diversity. 

Although not statistically significant, dominance of this species was confirmed when the reservoir rose from 253 m to 257 m. Dominance values for *Ma. humeralis* between stages 247 m and 257 m were not significantly different with the ST method (*P* = 0.5). When using HAT 24 h and HATet, the mean dominance of *Ma. humeralis* at stage 253 m increased significantly from 247 m. 

Monthly frequencies of other culicid species were correlated with the number of *Ma. humeralis* in all capture methods during the three flooding stages. In relation to the MA technique, no statistical difference was recorded in *Ma. humeralis *frequencies (*P* = 0.3) during stages 253 m and 257 m and for “other species” (*P* = 0.2) during stages 247 m and 253 m. 

No statistical difference was observed between mean temperature prior to flooding (23.3°C) and that following flooding stages 1 and 2 (24.7°C) when compared with *Ma. humeralis* frequency after analysis of all techniques (*P* > 0.1). The same occurred for mean precipitation, which was higher (95.2 mm) during the preflooding stage than subsequent periods (62.6 and 50.6 mm), but not statistically different. *Mansonia humeralis* showed greater population density during winter and spring (*P* > 0.1). 

## 4. Discussion 

 Mean richness and diversity values changed based on evaluation at each of the three reservoir levels. Immediately following initial flooding (flood stage 253 m), a 64% reduction occurred in the number of adult culicids, suggesting that many rearing sites were destroyed and immature forms did not survive the rising water. 

Increased *Ma. humeralis* abundance (from 3.1% at 247 m to 59.6% at 253 m and 53.8% at 257 m) suggests that this species benefitted from the flooding in relation to other culicids. This supports previous results following the flooding of the Tucuruí Reservoir in Amazonia [7], where high nuisance levels were recorded in humans and animals due to larger *Mansonia *populations (97.1%). 

*Mansonia* species were absent in forest fragments between the municipalities of Presidente Venceslau and Caiuá [18], near the study site sampled and in a similar ecological scenario. This contradicts our results in the preflooding stage, when an abundance value of 3.1% was recorded. The low frequency may be associated with the scarcity of macrophytes in the preflood water pools on the Paraná River floodplain. 

With damming and the expansion of macrophyte assemblages, distribution of *Ma. humeralis *increased in the study area and it became the dominant species. 

 Differences in Culicidae richness and diversity were substantial for flood stages 253 m and 257 m. Diversity, evaluated by ST, did not exhibit the same effect, possibly because this method was applied during a period of low mosquito activity. Nevertheless, evening crepuscular captures with ST and HATet produced similar results in relation to total mosquitoes captured, although they were used at different times. When analyzed in regard to richness and diversity prior to flooding, the capture method suggested variability between richness and diversity, and between diversity and dominance. However, hematophagic activity of *Ma. humeralis,* measured by HAT 24 h, best represented local fauna. The low capture rate of the MA method may be due to the random selection of shelters visited, while the higher rates observed with human presence are likely related to the anthropophilic behavior of *Ma. humeralis*. 

During immature collections along the São Domingos River (northern Paraná State), Lopes and Lozovei [19] concluded that culicids use forests adjacent to lakes as refuges. Their study area consisted of various forest fragments housing* Ma. humeralis,* while human settlements located within the dispersal radius of *Ma. humeralis *are permanent blood meal sources for females of these species. Thus, increased nuisance levels are expected during periods of higher *Ma. humeralis* activity. Similar observations were made in the Taquaruçu Reservoir, Paranapanema Bay [20]. More recently, Cruz et al. [21] found a predominance of *Mansonia* near the Madeira River hydroelectric plant in Amazonia, emphasizing the importance of monitoring activities in areas with new reservoirs. 

A strong positive correlation was recorded in the present study between artificial flooding and population levels of *Ma. humeralis*. In addition to being an indicator of macrophytes, this species can become a nuisance in areas surrounding a reservoir owing to its highly anthropophilic behavior, as was the case near the Tucuruí Revervoir [7]. 

Frequencies for other species were significantly different from mean values for *Ma. humeralis* in both flood stages, except for *Ma. humeralis *captured by MA at 253 m and 257 m. Findings with MA may be explained by the fact that the species studied does not have continuous distribution, exhibited by few individuals during the 2nd and 3rd stages and absent in stage 1. A low MA capture rate was observed for species other than *Ma. humeralis *at 247 m and 253 m stages. 

Overall, diversity and richness of mosquito fauna were substantially affected by flooding. However, there was a notable increase in the *Ma. humeralis* population with rising water levels. This species was present over the entire monitoring period, with peaks in winter and spring, a trend not observed for other culicids. Surface waters drain into the lake during flooding and enrich the reservoir with nutrients, thereby stimulating the proliferation of macrophytes [22] and favoring *Mansonia.* In addition, drainage channels may transport aquatic vegetation and disperse immature mosquitoes into other areas [3], making control measure a complex undertaking. 

Other investigations have emphasized the vulnerability of areas near the dam in providing favorable conditions for the spread of arboviruses, a concern reinforced by our results (Wanderley et al. [23]). 

*Ma. humeralis* was the primary focus of the present study due to the sharp increase in population dominance following flooding, which may generate a possible nuisance to the human population. However, a substantial amount of Culicidae relevant to public health was recorded, also reported in other research, including *Aedes scapularis* (Rondani) and *Anopheles albitaris* s.l. Lynch Arribálzaga [24, 25]. This research confirms the importance of monitoring Culicidae fauna. 

## 5. Conclusion 

The authors suggest that during the operational phase of this Hydroelectric Plant, local authorities should monitor culicid activity using sustainable entomological surveillance. 

## Figures and Tables

**Table 1:** Total number of Culicidae collected and mean monthly values of richness, diversity, and dominance of *Mansonia humeralis,* according to the collection technique at the JB site in the municipality of Presidente Epitácio, for the preflooding (level 247 m) and postflooding (253 m and 257 m levels) phases of the Parana River.

Taxonomic category	Level 247 m						Level 253 m						Level 257 m				Total	%
	MA	ST	HAT 24 h	HATet	Subtotal	%	MA	ST	HAT 24 h	HATet	Subtotal	%	MA	ST	Subtotal	%		
*Ae. scapularis*	134	701	6,463	1,304	8,602	38.8	6	186	88	12	292	3.7	23	38	61	10.9	**8,955**	29.1
***Ma. humeralis***	**—**	**280**	**339**	**76**	**695**	**3.1**	**3**	**1,182**	**2,887**	**682**	**4,754**	**59.6**	**2**	**299**	**301**	**53.8**	**5,750**	**18.7**
*Ma. titillans *	2	317	1,305	338	1962	8.8	2	326	245	120	693	8.7	1	15	16	2.9	**2,671**	8.7
*Cq. nigricans *	6	310	1,298	495	2,109	9.5	—	14	31	12	57	0.7	—	—	—	0.0	**2,166**	7.1
*An. (Nys.) albitarsis *s.l.	—	412	563	411	1,386	6.2	4	46	69	28	147	1.8	1	6	7	1.3	**1,540**	5.0
*Cq. hermanoi *	3	60	679	189	931	4.2	—	28	40	29	97	1.2	—	—	—	0.0	**1,028**	3.3
*An. galvaoi *	—	396	400	202	998	4.5	1	6	3	1	11	0.1	—	1	1	0.2	**1,010**	3.3
*Ad. squamipennis*	14	348	125	46	533	2.4	7	310	35	20	372	4.7	—	49	49	8.8	**954**	3.1
*An. triannulatus *	—	61	245	110	416	1.9	3	266	228	33	530	6.6	—	5	5	0.9	**951**	3.1
*Ps. (Jan.) albigenu *	3	61	675	74	813	3.7	—	3	28	—	31	0.4	—	1	1	0.2	**845**	2.8
*Ps. (Jan.) discrucians *	1	106	411	37	555	2.5	1	4	—	—	5	0.1	—	3	3	0.5	**563**	1.8
*Cx. (Aed.) amazonensis *	2	30	303	85	420	1.9	—	—	5	2	7	0.1	—	—	—	0.0	**427**	1.4
*Cx. (Mel.) ocossa *	50	6	30	—	86	0.4	160	90	40	8	298	3.7	3	12	15	2.7	**399**	1.3
*Cq. juxtamansonia *	1	78	211	68	358	1.6	—	4	13	9	26	0.3	—	10	10	1.8	**394**	1.3
*Ae. serratus *	22	7	237	71	337	1.5	—	—	5	2	7	0.1	—	—	—	0.0	**344**	1.1
*Cx. (Mel.) ribeirensis *	1	10	314	—	325	1.5		2	6	1	9	0.1	—	1	1	0.2	**335**	1.1
Total Culicidae (*)	**412**	**3,519**	**14,404**	**3,846**	**22,181**	**100.0**	**362**	**2,679**	**3,932**	**1,009**	**7,982**	**100.0**	**82**	**478**	**560**	**100.0**	**30,723**	**100.0**
Total of other species (except *Ma. humeralis*)	**412**	**3,239**	**14,065**	**3,770**	**21,486**	**—**	**359**	**1,497**	**1,045**	**327**	**3,228**	**—**	**80**	**179**	**259**	**—**	**24,973**	**—**
Richness (mean monthly value)	**8.5**	**13.5**	**25.8**	**16,2**	**—**	**—**	**5.5**	**10.1**	**11.3**	**6.3**	**—**	**—**	**1.9**	**4.7**	**—**	**—**	**—**	**—**
Diversity (mean monthly value)	**2.4**	**2.4**	**3.7**	**2.8**	**—**	**—**	**1.6**	**1.8**	**1.9**	**1.3**	**—**	**—**	**0.6**	**1.4**	**—**	**—**	**—**	**—**
Dominance of *Ma. humeralis* (mean monthly value)	**—**	**8.2**	**3.3**	**2.1**	**—**	**—**	**1.7**	**44.4**	**59.8**	**60.2**	**—**	**—**	**2.2**	**25.2**	**—**	**—**	**—**	**—**

*Amounts including mosquito species with 1.0% or less specimens: *An. rondoni, Cx. (Culex) *spp. (1.0%);* Cx. (Mel.)* Melanoconion section, *Ps. (Jan.) ferox *(0.7%);* An darlingi *(0.6%);* Cx. (Mel.). *Atratus group (0.5%); *Cx. (Mel.) idottus *(0.4%); *An. parvus, Cq. shannoni, Wy. melanocephala* (0.3%);* Cx. (Mel.) *Intrincatus group, *Ma. amazonensis *(0.3%); *An. (Nys.) *spp*., Cq. albicosta, Cx. (Cux.) bidens, Cx. (Cux.) declarator, Cx. (Cux.) *Coronator group,* Cx. (Mel.) clarki, Cx. (Mel.) oedipus, Ma. wilsoni, Sa. glaucodaemon, Ur. geometrica* (0.1%); *Ae. (Ste.) albopictus, Ae. fulvus, Ae. hastatus/oligopistus, Ae. nubilus, Ae. serratus/nubilus, Ae. aenigmaticus, An. braziliensis, An. deaneorum, An. evansae, An. strodei, Cq. chrysonotum/albifera, Cq. venezuelensis, Cx. (Cux.) camposi, Cx. (Cux.) chidesteri, Cx. (Cux.) coronator, Cx. (Cux.) maxi, Cx. (Cux.) mollis, Cx. (Cux.) quinquefasciatus, Cx. (Mel) *spp*., Cx. (Mel.) aureonotatus, Cx. (Mel.) bastagarius, Cx. (Mel.) contei, Cx. (Mel.) delpontei, Cx. (Mel.) dunni, Cx. (Mel.) eastor, Cx. (Mel.) flabellifer, Cx. (Mel.) *Pilosus group,* Cx. (Mel.) pavlovsky, Cx. (Mel.) pilosus, Cx. (Mel.) rabelloi, Cx. (Mel.) *sp*. * of Atratus group,* Cx. (Mel.) theobaldi, Cx. (Mel.) vaxus, Cx. (Mel.) zeteki, Cx. (Mel.) adamesi, Li. durhamii, Li. flavisetosus, Ma. indubitans, Ps. (Gra.) *sp*., Ps. (Jan.) albipes, Ps. (Pso.) ciliata, Ps. (Gra.) confinnis, Ur. apicalis, Ur. lowii, Ur. mathesoni, Ur. pulcherrima, Ur. *spp*.,* and* Wy. chalcocephala/roucouyana *(<0.1%).
